# Development of an evaluation tool for geriatric rehabilitation care

**DOI:** 10.1186/s12877-019-1213-0

**Published:** 2019-08-02

**Authors:** Meriam M. Janssen, Willeke Vos, Katrien G. Luijkx

**Affiliations:** Tranzo Department, Scientific Center for Care and Welfare, Tilburg University, PO Box 90153, 5000 LE Tilburg, The Netherlands

**Keywords:** Geriatric rehabilitation care (GRC), Quality improvement, Evaluation, Realist evaluation, Mechanisms, Context, Outcomes, GRC evaluation tool

## Abstract

**Background:**

Geriatric rehabilitation care (GRC) is short-term and multidisciplinary rehabilitation care for older vulnerable clients. Studies were conducted about its effects. However, elements that influence the quality of GRC have not been studied previously.

**Methods:**

In this study realist evaluation is used to find out which are the mechanisms and outcomes and which (groups of) persons are the context for GRC, according to GRC professionals. The mechanisms, outcomes and context of GRC were explored in three consecutive phases of qualitative data gathering, i.e. individual interviews, expert meeting, and focus groups.

**Results:**

Eight mechanisms — client centeredness, client satisfaction during rehabilitation, therapeutic climate, information provision to client and informal care givers, consultation about the rehabilitation (process), cooperation within the MultiDisciplinary Team (MDT), professionalism of GRC professionals, and organizational aspects — were found. Four context groups—the client, his family and/or informal care giver(s), the individual GRC professional, and the MDT—were mentioned by the respondents. Last, two outcome factors were determined, i.e. client satisfaction at discharge and rehabilitation goals accomplished.

**Conclusions:**

In order to translate these insights into a practical tool that can be used by MDTs in the practice of GRC, identified mechanisms, contexts, and outcomes were visualized in a GRC evaluation tool. A graphic designer developed an interactive PDF which is the GRC evaluation tool. This tool may enable MDTs to discuss, prioritize, evaluate, and improve the quality of their GRC practice.

**Electronic supplementary material:**

The online version of this article (10.1186/s12877-019-1213-0) contains supplementary material, which is available to authorized users.

## Background

Internationally, rehabilitation care for older adults is provided in different settings, such as in hospitals (rehabilitation ward, long-term care hospitals, community hospitals), skilled nursing facilities, care homes or in ambulatory settings [1]. In the Netherlands, geriatric rehabilitation care (GRC) is provided in nursing homes. GRC is defined as multidisciplinary rehabilitation care for older and vulnerable clients who were hospitalized and need short-term rehabilitation in a nursing home. Five diagnoses are distinguished; stroke, elective orthopedics, trauma, amputations, and other disorders [2]. GRC is provided by a MultiDisciplinary Team (MDT). The elderly care physician has the final medical responsibility within the MDT and nurses provide care. In addition, clients rehabilitate in therapy given by specialized paramedic professionals such as occupational therapists, physiotherapists, speech therapists, psychologist and dietician. Rehabilitation care is focused on recovery and returning home [3–5].

In the Netherlands, 145 organizations provide GRC. In 2015 45.000 clients received GRC of whom 80% could return home after treatment [3]. In 2017 2% of the clients received GRC because of an amputation, 18% because of a stroke, 14% for elective orthopedics, 30% for trauma, and 37% for other disorders [6]. Average length of stay was 45 days [3], and specified per diagnosis it was 65 days for amputation, 47 days for stroke, 28 days for elective orthopedics, 45 days for trauma, and 39 days for other diseases [6].

Literature about evaluations of GRC mainly focuses on its effects in general or for specific groups of clients. It was found that compared to older adults in a general medical ward setting older adults receiving specialized GRC were more likely to return home, had lower (in-hospital) mortality rates [7–10], had a reduced length of a hospital stay [11], were less likely to have cognitive or functional decline [7], and improved on independence in ADL [12]. Moreover, older adults seemed to benefit more from multidisciplinary programs with an exercise program than care as usual with an exercise program, that is, they improved more on physical tests and performance and they were more likely to return home instead of being discharged to a nursing home [11].

Effects were also found for specific groups of clients. Better functional outcomes, increased odds of going home, and a reduced length of stay were found for stroke clients who rehabilitated in a multidisciplinary therapy-based setting, compared to a general medical setting [13]. Also, clients in specialized orthopedic geriatric rehabilitation improved better for activities in daily life (ADL), returning home, and mortality, compared to care as usual [14]. Evaluation of a geriatric rehabilitation program aimed to improve the quality of care found that clients with traumatic injuries were more independent in ADL, were more likely to be discharged to home, and had a shorter length of stay one year after the implementation of the program. Moreover, stroke clients were more independent in ADL one year after the implementation of the program [12]. Moreover, for orthopedic rehabilitation, it seemed that specialized care helped successful geriatric rehabilitation for total joint replacement [15] and specifically for clients who suffered from a hip fracture, geriatric rehabilitation improved the ability of independent living on the short term, which enlarged the possibilities for discharge to home [16].

GRC is existing for a long time, but can always be improved. At the start of the specialized field of GRC in the Netherlands, funding was provided through the General Special Medical Expenses Act (AWBZ), and it was a kind of long-term residential care. Since 2013, funding is provided through healthcare law [2, 17]. Since then, GRC aims at recovery and is more result-driven. GRC works with diagnosis-treatment-combinations. In a diagnosis-treatment-combination, each rehabilitation effort has a beginning and an end (and is thus short-term care) and is aimed at rehabilitation and returning home. Since GRC has been funded through healthcare law, budgets have stayed similar but more clients were treated in GRC and more clients returned home after a shorter length of stay [18–21].

Many studies were conducted on the effects of GRC. Moreover, since GRC is funded through the healthcare law, emphasis is put on the effects of GRC in the Netherlands. However, to our best knowledge, the way the outcomes and care processes in GRC can be improved, leading to GRC quality improvement, has not been studied before. If GRC professionals regularly reflect on and discuss, evaluate, and improve the care processes and outcomes of their daily GRC practice, quality of GRC might be positively influenced. Therefore, it is important to increase knowledge about which elements are able to influence the quality of GRC. In GRC the setting, i.e. the context, is multidisciplinary; clients receive care from nurses and elderly care physicians. In addition, clients rehabilitate in therapy given by specialized paramedic professionals. An evaluation approach that takes this context into account is realist evaluation. Other evaluation approaches only look at ‘does something work’, i.e. do mechanisms lead to a certain outcome, or not? Realist evaluation is an approach that looks at ‘what works, for whom in what circumstances and how’ for complex and multidisciplinary care settings, [22–24] and is likely to suit the complex multidisciplinary care setting of GRC. Realist evaluation is about finding out how things work, and looks at the interplay between context and mechanisms, and thus how a context triggers (or interferes with) mechanisms, leading to an outcome. Getting insight into these interactions can explain why outcomes might differ, due to a positive, neutral, or negative interaction between mechanisms and context in different care settings [23, 25]. Outcomes therefore depend on both the mechanism and the context [23].

In this study we aim to find out which are the elements with which the quality of daily GRC practice can be evaluated and improved. And, thus, which are the mechanisms, contexts, and outcomes of GRC. Moreover, we aim to translate this theoretical knowledge into a practical GRC evaluation tool that has added value for and is usable in GRC practice, i.e. a tool which will enable MDTs to reflect on their GRC, and thus to discuss, prioritize, evaluate, and improve the quality of their GRC practice. Therefore, this study was conducted in co-creation with GRC professionals from a multidisciplinary perspective, to include nurses, elderly care physicians, and paramedic professionals. The research questions of this study are: (1) what are the mechanisms, which (groups of) persons are the context, and what are the outcomes of GRC in the Netherlands by which GRC-professionals are able to discuss, prioritize, evaluate, and improve the quality of GRC; and (2) how can these mechanisms, contexts, and outcomes be translated into a GRC evaluation tool that can be used to improve the quality of daily GRC practice?

## Methods

A qualitative study, based on realist evaluation, was conducted with GRC professionals working in three different organizations. This study was approved by the Ethical Review Board of the Tilburg School of Social and Behavioral Sciences of Tilburg University (registration number EC-2017.52).

### Selection of organizations

The Academic Collaborative Center (ACC) Older Adults of Tranzo, Tilburg University, in the Netherlands, aims to advance care for older adults towards person-centered care through equal cooperation between science and practice. The ACC Older Adults aims to translate theoretical knowledge into practical tools, in co-creation with (long-term) care organizations, in order to ensure that the knowledge from scientific research is usable for professionals working in these organizations. The current study was explained during a meeting with the contact persons of the 11 participating care organizations. Of the nine organizations offering GRC, three organizations of two different regions in the Netherlands were enthusiastic to participate in the study. In the results section, the three participating GRC organizations are described.

For each organization a meeting was organized between the GRC manager and the researcher. The study was explained in more detail as well as the needed effort that would be required if the organization decided to participate. Subsequently, in each organization, the GRC steering group and/or GRC manager decided whether their GRC organization would participate in the study. We valued the importance of this process because of the support and commitment required for the study. All three GRC organizations decided to participate.

The executive boards of the organizations were informed about the study and asked for written approval, which was also given.

### Selection of participants

The GRC manager for each participating organization was asked to select two GRC professionals who were able to reflect critically on their GRC practice. GRC encompasses multidisciplinary care, provided by nursing staff, elderly care physicians, and paramedic professionals. Therefore, the GRC managers were asked to select two different disciplines per organization. The respondents were approached face-to-face by the manager. Also, they were thus selected intentionally, not at random [26]. All respondents approached by the GRC manager participated.

Respondents and researcher did not know each other beforehand. Respondents were only told that they were to be interviewed by a researcher working at the ACC Older Adults of Tranzo, Tilburg University.

All respondents received an information letter about the study. If a respondent decided to participate, a written informed consent letter was signed.

### Data collection and analysis

Data collection took place between July and November 2017 and was done by the first author (MJ). She is very experienced in conducting qualitative research and worked as a postdoc researcher in this project. Data collection was done in three consecutive phases.

First, per respondent, an individual interview was conducted about her GRC practice in general and also more specifically about the mechanisms, contexts, and outcomes in GRC. In Additional file 1, the topic guide is included. Only the respondent and researcher were present during the interview. The topic list was drafted by the researcher and first author (MJ). After a discussion session with the other two authors (WV, KL) it was made final. Each semi-structured interview lasted 60 to 90 min. All interviews were audiotaped and field notes were made by the first author. A detailed and anonymized conversation report was made and sent to the respondent in order to conduct a member check.

Two researchers (MJ and KL) independently from each other analyzed two distinct interviews. In a discussion session, consensus was reached about the main topics and the subtopics per main topic, which were derived from the interviews. After consensus was reached, the researcher and first author (MJ) analyzed the remaining interviews.

Second, an expert meeting with the respondents of the individual interviews plus one extra GRC professional was organized which lasted 120 min. Besides the respondents, two extra researchers (KL and WV) were present during the expert meeting. The results of the individual interviews were explained. All participants confirmed the findings. Based on the findings of the interviews, the mechanisms, contexts, and outcomes of GRC emerged. Respondents were asked to concretize each mechanism, which was a first step in defining each mechanism. In Additional file 2, the agenda of the expert meeting including the topics that needed to be discussed, is presented. A conversation report of the concretization per mechanism was made and sent to the respondents to conduct a member check.

Third, for each participating organization, one focus group was conducted with the respondents of phase 1 and 2, complemented with a GRC manager. Only the respondents and the researcher were present. Each focus group lasted 60 to 90 min. All focus groups were audiotaped. The goal of these focus groups was to finalize the definitions of the mechanisms in order to be able to develop the GRC evaluation tool. Discussion point for the focus groups was a concretization per mechanism. The researcher and first author (MJ) drafted a definition of each mechanism, based on the output of the individual interviews and expert meeting (phase 1 and 2). This first draft version was discussed with the respondents of the first organization and respondents could complement and improve it. After the first focus group a conversation report and improved definitions of the mechanisms (second draft) were made and sent to the respondents for a member check. This second draft was discussed in the focus group with the respondents of the second organization, which was also improved on by the respondents of the second focus group into a third draft and was sent for a member check. Last, the third draft was discussed in the focus group with the respondents of the third organization and again it was improved. After a final member check with the respondents of the focus group of the third organization the last draft was made final.

## Results

### GRC organizations

Three GRC organizations participate in this study. All three organizations provide specialized GRC.

The first organization is an independent GRC organization which has six departments in five locations with in total 119 beds and 180 GRC professionals (113 fulltime-equivalent).

The second and third organizations are part of a larger elderly care organization, providing GRC, home care, long term care, and palliative care. The second organization has 20 locations. In total, 2832 persons (1361 fulltime-equivalent) work in this organization, in management, staff, supporting services and care. In total, 1736 professionals (875 fulltime-equivalent) work in care, of whom 89 professionals (63 fulltime-equivalent) work in GRC. At two locations, GRC is provided in six departments with in total 89 beds.

The third organization has eight locations. About 1850 professionals (1030 fulltime-equivalent) work in this organization. In one location, at three departments, GRC is provided with in total 51 beds. The fulltime-equivalent of the elderly care physicians and nurses is 25.1. Although they work in MDTs, the fulltime-equivalent of the paramedic professionals was not specified, because they work at GRC as well as other departments in this organization.

### Respondents

In total, 10 respondents from three GRC organizations of two regions in the Netherlands participated in this study. All respondents were female.

First, six individual interviews with three nurses, one elderly care physician, one speech therapist, and one occupational therapist were conducted, two per participating organization. Each respondent was interviewed at her workplace.

Second, seven respondents participated in the expert meeting organized at Tranzo, Tilburg University.

Third and last, three focus groups (one per participating organization) were conducted with nine respondents, at the workplace of the respondents.

See Table 1 for a description of the participants for phases 1–3 of data collection.

**Table 1:** Description of participants per phase of data collection

Phase of data collection	Phase 1: 6 individual interviews about GRC in general and also specifically about the mechanisms, context, and outcomes	Phase 2: 1 expert meeting, first definition of the mechanisms, context, and outcomes of GRC	Phase 3: 3 focus groups (1 per participating organization) to finalize the definition of the mechanisms, context, and outcomes to develop the practical GRC evaluation tool
Number of females	6	7	9
Occupational function:			
-Nurse	*N* = 3	*N* = 3	*N* = 3
-Occupational therapist	*N* = 1	*N* = 1	*N* = 1
-Speech therapist	*N* = 1	*N* = 1	*N* = 1
-Elderly care physician	*N* = 1	*N* = 2	*N* = 1
-Coordinating nurse			*N* = 1
-GRC manager			*N* = 2

This current study was conducted according to the approach of realist evaluation. In the data gathering we were looking for the mechanisms, contexts, and outcomes of GRC. These are explained below. After this explanation the cohesion between the mechanisms, contexts, and outcomes will be clarified with an example of the interaction between a mechanism and a context group leading to a certain outcome.

### Mechanisms

Eight mechanisms were found that can be divided into three levels of a GRC organization. First, three mechanisms are on the level of the client; client centeredness, client satisfaction during rehabilitation, and therapeutic climate. Second, four mechanisms are on the level of the GRC professionals and the MDT; information provision to client and informal care givers, consultation about the rehabilitation (process), cooperation within the MDT, and professionalism of GRC professionals. Third and last, one mechanism is on the GRC organizational level, that is, organizational aspects.

Thereafter, in co-creation with the respondents, the eight mechanisms were defined based on daily GRC practice during the expert meeting (phase 2) and focus groups per organization (phase 3).

Table 2 shows an overview of the mechanisms and the definition of each mechanism.

**Table 2:** Overview of the mechanisms and definition of each mechanism

Mechanism	Definition
Client-centeredness	During the entire rehabilitation: - GRC professionals are continuously in conversation and agreement with the client - Goals are determined together with the client and suit the vulnerability of the client - The MDT regularly evaluates the rehabilitation plan with the client and the informal care givers - Preferably, the client is present during the MDT-consultation about his rehabilitation (process)
Client satisfaction during rehabilitation	- The client is continuously involved in his rehabilitation process and all involved GRC professionals listen carefully to his input - Client knows which GRC professional he can ask questions of or share worries about his rehabilitation - The client is regularly asked about his satisfaction - Client feels safe and treated with respect
Information provision to client and informal care givers	- Before admission, the client is well informed about what he can expect during GRC and what is expected from him concerning the therapeutic climate - During admission, information is given by one GRC professional and it suits the vulnerability of the client - The client is continuously informed about the progress and/or decline of his rehabilitation - Elderly care physician and client regularly talk about (the progress of) the rehabilitation process - Informal care givers are involved in the therapy - Client knows when therapy sessions takes place (even if the date and/or time of the therapy session changes) - The provisional discharge date is determined and communicated as soon as possible - Client is informed about the ‘things to organize’ concerning discharge
Consultation about rehabilitation (process)	- Client and involved GRC professionals subscribe the rehabilitation goals - The progress of each rehabilitation process is regularly discussed - Registrations in the Electronic Client Dossier (ECD) are always up-to-date and all involved GRC professionals are aware of the up-to-date situation of a client - All MDT-consultations contribute to (1) the rehabilitation (outcome) of an individual client, or (2) the process of GRC, or (3) mutual cooperation
Cooperation within MDT	- All involved GRC professionals can consult each other easily - The elderly care physician is in charge of the rehabilitation process - Expertise of all MDT professionals is taken seriously and all GRC professionals are equal to each other - MDT professionals work in mutual respect and they trust each other; there is a pleasant way of working together - MDT professionals can learn from each other. Feedback can be given and received in a constructive and safe way
Therapeutic climate	- Each client trains 24/7 according to the principle ‘everything is rehabilitation’ (=therapeutic climate) - All involved GRC professionals stimulate the self-reliance of each client - Prior to admission, the client is aware of the therapeutic climate in GRC and he has a positive attitude about it - Rehabilitation goals are continuously coordinated with the client - Informal care givers are explicitly involved in the therapy and are aware that they can practice with the client
Professionalism of GRC professionals	- GRC professionals are enthusiastic and motivated to work in GRC - GRC professionals proactively suggest which education, which contributes to their GRC-professionalism, they would like to follow - At MDT level, there is insight into what expertise is needed and which expertise is (not) present
Organizational aspects	Admission: - For referrers to GRC (mostly hospitals) it is clear which clients are able to rehabilitate and can be referred to GRC - The elderly care physician has the final judgment whether a client can be rehabilitated and can be included in GRC During rehabilitation: - All involved MDT professionals have enough time to gain insight into the health situation of the client and to deliver the right care, appropriate to the goals and resilience of the client - One GRC manager decides for GRC and the MDT, even if more managers are involved. Decisions are made with the aim of realizing a good rehabilitation outcome and a satisfied client - New initiatives and innovation that can improve GRC can be tried Discharge: - At discharge, the client is referred to a living situation that suits him best; home, a nursing home, or a home that combines independent living with care

### Context

Four context groups—the client, his family and/or informal care giver(s), the individual GRC professional, and the MDT—were mentioned by the respondents. These (groups of) persons interact positively, neutrally, or negatively with one or more mechanisms, which will (not) contribute to a positive outcome.

### Outcomes

According to the respondents, two outcome factors for the clients are important, client satisfaction at discharge and rehabilitation goals accomplished. Respondents explained that to find out whether a client is satisfied at discharge, each client completes a small questionnaire. If necessary, a short interview will take place between the client and a GRC professional to gain more insight into the satisfaction and points for improvement.

As regards ‘rehabilitation goals accomplished’, at admission the rehabilitation goals are formulated in agreement between the client and the MDT and adjusted during the rehabilitation if necessary. Most of the time the goals concern general daily activities such as washing yourself and getting dressed in the morning, preparing drinks and meals, and going to bed in the evening, and thus being able to live as independently as possible. It is important that the goals suit the health situation and vulnerability of the client. If the rehabilitation goals are accomplished, the outcome of GRC can be seen as successful according to the respondents.

### An example of the interaction between a mechanism and a context group leading to a certain outcome

An example can clarify the way in which a mechanism and a context group interact with each other and lead to a certain outcome. Respondents defined the mechanism ‘therapeutic climate’ as follows: a therapeutic climate is an environment in which a client is given the opportunity to work on his recovery and is stimulated to train 24/7 according to the principle ‘everything is rehabilitation’. The client does as much as possible himself, and help is only provided for things that the client cannot do independently. It means that all activities from getting up in the morning until going to sleep in the evening, such as getting dressed and preparing meals, as well as the therapy sessions, are part of the rehabilitation process. A client (context group) with a positive attitude toward therapeutic climate will decide to rehabilitate according to the principle of therapeutic climate, and thus tries to do as much as possible independently. This will lead to a positive interaction between the mechanism and context, because this will stimulate the rehabilitation and a positive rehabilitation outcome. However, another client might have a (more) negative attitude towards therapeutic climate. He might only make an effort during the therapeutic sessions for rehabilitation, and might expect that he will be taken care of during daily care activities, instead of feeling the need that he must do as much as possible independently. This might lead to a negative interaction between the mechanism ‘therapeutic climate’ and the client (context), which might lead to a less successful outcome.

Moreover, besides the client as a context group, the individual GRC professional also interacts as a context group with the mechanism ‘therapeutic climate’. An individual GRC professional who stimulates a client in doing as much as possible independently, and thus stimulates the client to rehabilitate according to the principle of therapeutic climate, will interact positively with the mechanism ‘therapeutic climate’. This positive interaction will help to reach the outcome of ‘accomplishing rehabilitation goals’.

### Development of the GRC evaluation tool

As mentioned before, the ACC Older Adults aims to translate theoretical knowledge into practical tools for (long-term) care organizations, and in co-creation with care professionals. The aim of this study was to translate the theoretical findings (i.e. the mechanisms, contexts, and outcomes of GRC according to the approach of realist evaluation) into a practical tool with which GRC professionals are able to reflect, discuss, prioritize, evaluate, and improve the quality of their daily GRC. The development of the GRC evaluation tool was done in two phases, i.e. first the content and second the design.

The content of the GRC evaluation tool, and thus the mechanisms and its definitions, the contexts, and the outcomes, was gathered in the individual interviews, expert meeting and focus groups. For designing the GRC evaluation tool, the mechanisms and its definitions, the contexts, and the outcomes were discussed with a graphic designer. Moreover, the way a mechanism and context can interact, leading to a certain outcome, was also discussed with the graphic designer. It was necessary for her to understand the theoretical findings of this study and the setting in which the tool will be applied, in order to be able to design a GRC evaluation tool that suits the GRC practice.

Based on this input, the graphic designer designed an interactive PDF, that is, the GRC evaluation tool, with which GRC professionals are able to discuss, prioritize, evaluate, and improve the quality of their daily GRC practice.

### How does the GRC evaluation tool work?

Working with the GRC evaluation tool goes as follows. It is important that all members of a MDT are involved. First, all members of a MDT write down individually which mechanism he/she chooses to improve. Second, plenary each MDT member shares which mechanism he/she chooses. The mechanism which is chosen most, will be selected for improvement. Third, in a plenary discussion, the MDT reflects and discusses what goes well and what can be improved for the chosen mechanism. By clicking on the chosen mechanism, the definition of this mechanism (see Table 2) is shown. This enables a MDT to discuss what goes well for the chosen mechanism and what can be improved. The interaction of each context group with the chosen mechanism, leading to a certain outcome, can also be discussed. Fourth, specific actions for improvement are formulated, based on ‘what can be improved for the chosen mechanism’. Moreover, the person who is responsible to complete a certain action is also formulated. The GRC evaluation tool is interactive, which means that the chosen mechanism, what goes well and what can be improved, and the specific actions for improvement can be filled in into the GRC evaluation tool. This will help MDTs to reflect on their care processes and outcomes and to improve the quality of their daily GRC practice.

**Fig. 1:**
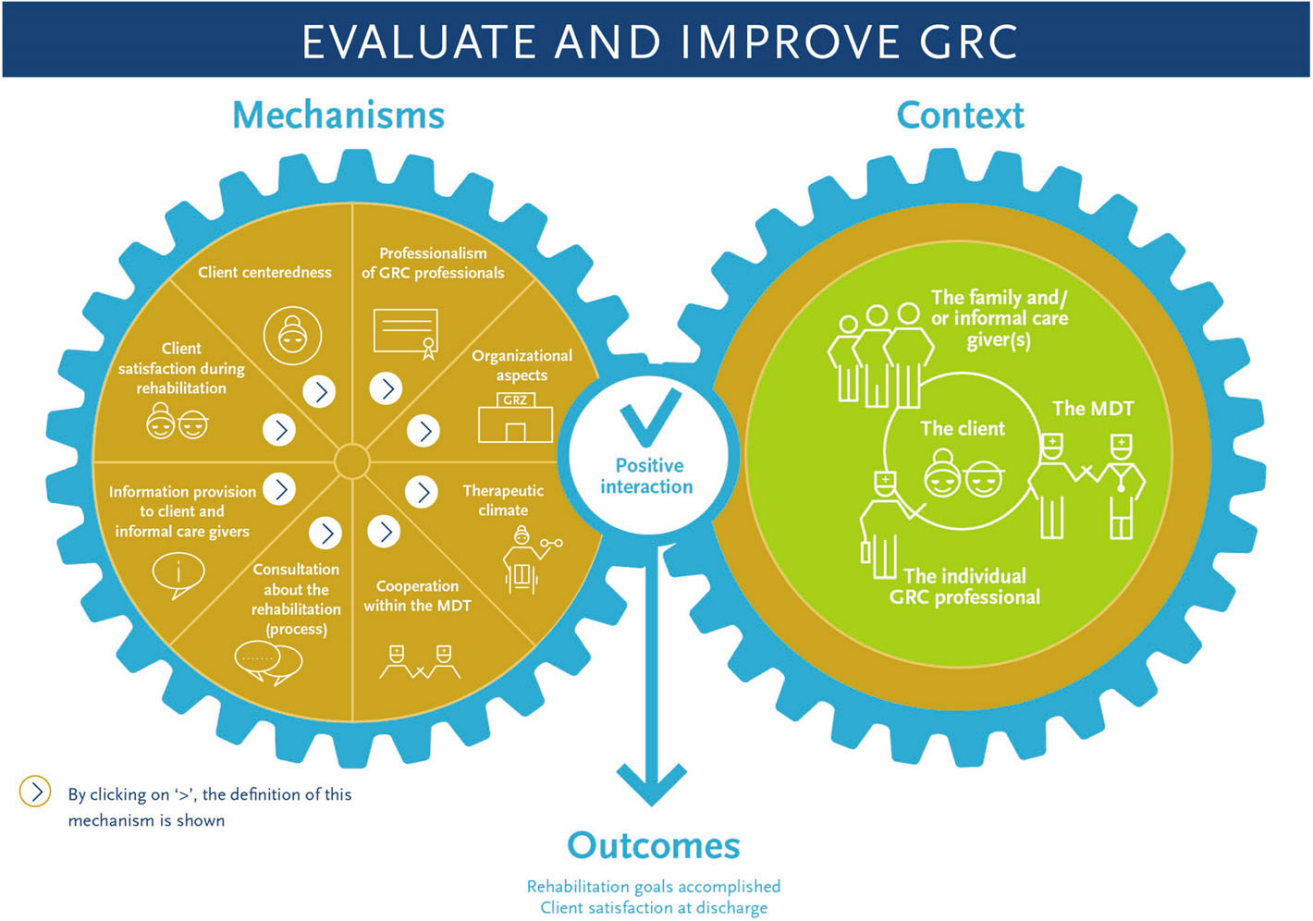
The GRC evaluation tool

Figure 1 shows the front page of the GRC evaluation tool.

## Discussion

Two research questions were formulated for this study: (1) what are the mechanisms, which (groups of) persons are the context, and what are the outcomes of GRC in the Netherlands by which GRC professionals are able to discuss, prioritize, evaluate, and improve the quality of their GRC and (2) how can these mechanisms, contexts, and outcomes be translated into a GRC evaluation tool that can be used to improve the quality of daily GRC practice? Eight mechanisms, that is, client-centeredness, client satisfaction during rehabilitation, therapeutic climate, information provision to client and informal care givers, consultation about the rehabilitation (process), cooperation within the MDT, professionalism of GRC professionals, and organizational aspects were found. Also, four context groups, that is, the client, his family and/or informal care giver(s), the individual GRC professional, and the MDT, and two outcomes, client satisfaction at discharge, and rehabilitation goals accomplished, were found.

The way one or more of the context groups interact (positively, neutrally, negatively) with one or more mechanisms leads to a successful outcome, or not. Based on the approach of realist evaluation, the question of whether a certain mechanism will lead to a certain outcome is not the only factor. Inclusion of the context is also important because (a group of) persons, that is, the context, decide whether they will interact with a mechanism, which as a result will determine whether a positive outcome can be reached (for a clarification, see the example in the results section). By taking the context into account, the realist evaluation questions ‘what works, for whom in what circumstances and how’ for complex and multidisciplinary care settings can be answered [22–24].

In a PhD-study, conducted within the ACC Older Adults, a model was developed with which integrated care interventions can be evaluated [27, 28]. This model was also developed according to the realist evaluation approach and, therefore, it is called the COMIC model, the Context, Outcomes and Mechanisms of Integrated Care interventions model. The innovative aspect of this model is that an evaluation of integrated care interventions is based on the integrated care intervention (mechanisms) itself, the context in which the intervention takes place, and the interplay between intervention and context. These elements determine the outcomes of the intervention and thus the effectiveness and/or satisfaction with a certain care intervention. The mechanisms and context of the COMIC model are based on two existing theoretical models, namely the Chronic Care Model and the Implementation Model [28–31]. The use of both models is widespread [32–34]. The outcomes are based on the six dimensions of quality of care as formulated by the World Health Organization [28, 29].

If we compare the mechanisms and contexts of the COMIC model [27, 28] and the mechanisms and contexts of the GRC evaluation tool developed in this current study, we can see that similar terms were found. An overview of the mechanisms and contexts of the COMIC model and the GRC evaluation tool is given in Table 3. This insight, that similar terms for mechanisms and contexts in two different studies were found, strengthens the scientific basis of the GRC evaluation tool. Moreover, it might indicate that the GRC evaluation tool might be easily adaptable for other fields than GRC in geriatric care.

**Table 3:** Mechanisms and context of the COMIC model and the GRC evaluation tool

COMIC model	GRC evaluation tool
Mechanisms	
Patient (centeredness) and decision support	Client centeredness
Satisfaction	Client satisfaction during intramural rehabilitation
Self-management support and accessibility	Provision of information to the client and family
Organizational context and decision support	Consultation about rehabilitation (process)
Community	Cooperation within the multidiciplinary team
Delivery system design	Therapeutic climate
Innovation	Professionalism of GRC professionals
Health system context and organizational context	Organizational aspects
Context	
Patient	Client
Social context	Family and/or informal care giver(s)
Individual professional	Individual GRC professional
Organizational context	Multidiciplinary team

### Strengths and limitations

A strength of this study is that the professionals working in GRC formulated the mechanisms, contexts, and outcomes of GRC. The content of the GRC evaluation tool is thus formulated by and for the professionals who are intended to use the evaluation tool, in a saturated co-creation between science and practice. This increases the likelihood that GRC professionals recognize the content of the GRC evaluation tool and that the tool suits their daily care practice, which might increase the chance for a good and structural implementation.

A limitation is that only 10 respondents were interviewed in the three consecutive phases. This is due to the length of the study, which lasted 15 months. However, a good mix of respondents, specifically, nurses, paramedic professionals, elderly care physicians, a coordinating nurse, and a GRC manager from three different GRC organizations, was selected. They were all able to reflect critically on GRC and the care their GRC organization provides and what is needed to improve this care. All respondents provided extensive input about the mechanisms, contexts, and outcomes and the development of the GRC evaluation tool which was in line with each other’s’ input. Data saturation was reached despite a low number of participants.

Also, older adults in GRC were not interviewed in this study. Interviewing them would have provided insight into important elements according to clients. The client perspective is important to GRC professionals as they reported client satisfaction as one of the two outcomes. During the evaluation of the GRC evaluation tool we will take the client perspective into account.

A third and last limitation compromises the generalizability of the mechanisms, contexts, and outcomes, that is, do they relate to Dutch GRC care? A strength in this, however, is that data gathering was done in two different regions in the Netherlands. In addition, implementation of the evaluation tool has already started. The mechanisms, contexts, and outcomes of GRC as they are formulated in the evaluation tool are presented to several MDTs of the three participating organizations. The GRC professionals working in these teams recognized the formulated mechanisms, contexts, and outcomes, which seems to be another strength of this study.

### Implications for future research

More research is needed. In fall 2018, the GRC evaluation tool, its content as well as the experiences of working with the tool, will be evaluated. Based on the results of the evaluation the GRC evaluation tool will be developed further.

Moreover, in this study, the mechanisms, contexts and outcomes were mentioned by GRC professionals, and thus by the health care providers. The respondents in this study, i.e. GRC professionals, formulated the two outcomes, i.e. client satisfaction at discharge and rehabilitation goals accomplished. For future research, it would be of added value if we could define and measure the outcomes. Moreover, it would be of added value to take the perspective of the client into account, and thus that clients could also give meaning to the outcomes and could (co) define the outcomes. Patient-reported outcome measures (PROMs) measure patients’ perspectives on health outcomes [35] and can be used to define the outcomes according to the GRC clients. This is a second implication for future research.

## Conclusions

Insight into the mechanisms, contexts, and outcomes of GRC was gathered, based on realist evaluation. In order to translate these theoretical findings into a practical tool that can be used by GRC professionals, a graphic designer made the interaction between the mechanisms, contexts, and outcomes visual and developed an interactive PDF, i.e. the GRC evaluation tool. This tool may enable GRC professionals to discuss, prioritize, evaluate, and improve the quality of their daily GRC.

## Additional files

Additional file 1: Topic guide individual interviews GRC professionals. (DOCX 17 kb)

Additional file 2: Agenda expert meeting including topics to be discussed. (DOCX 15 kb)

## Data Availability

The dataset consists of qualitative Dutch data. All respondents were informed that the data gathered would only be used and read by the researchers and will not be given to third parties. Therefore, the datasets generated and/or analyzed during the current study are not publicly available.

## Abbreviations

ACC: Academic Collaborative Center
ADL: Activities in daily life
COMIC model: Context, Outcomes and Mechanisms of Integrated Care interventions model
GRC: Geriatric rehabilitation care
MDT: MultiDisciplinary Team
PROMs: Patient-reported outcome measures
