# Supplementary material for: Development of an evaluation tool for geriatric rehabilitation care
Source: BMC Geriatr. 2019 Aug 2;19:206. doi: 10.1186/s12877-019-1213-0 (PMC6679545; doi:10.1186/s12877-019-1213-0)
Supplement: Supplementary file 1 — Topic guide individual interviews GRC professionals. (DOCX 17 kb) [file 12877_2019_1213_MOESM1_ESM.docx]

**Additional file 1: Topic guide individual interviews GRC professionals**

**Introduction:**

- Thank participant for their participation
- Explain aim of the interview, i.e. ask input for developing an instrument to evaluate and improve GRC
- Obtain informed consent, including consent for audiotaping and transcribing the interview
- Provide general information, e.g. duration of interview, possibility of taking a break
- Ask if participant has any questions
- Repeat the aim of the interview
- Start the interview and switch the audio on!

**Interview questions:**

- Can you introduce yourself? Name, age, education, number of working years in GRC, at which organization(s), with which client population?
- How do you like working in the GRC? Why did you decide to start working in GRC? Can you illustrate your satisfaction with working in the GRC using a story/stories?
- What is the average length of stay in this department?
- During the admission of a new client:
  - What happens?
  - Who are involved? In which manner?
  - Which information from the client do you need?
  - When is the MultiDisciplinary Team (MDT), i.e. the elderly care physician, nurses and paramedics, satisfied? How do the different disciplines cooperate? When is the MDT not satisfied? Can you explain with some examples?
  - When is the client satisfied? How do you know that your client is satisfied? Examples?
- During the rehabilitation in the GRC:
  - What happens?
  - Who are involved? In which manner?
  - Which information from the client do you need?
  - Can you explain with some examples what goes well? And what does not go well?
  - When is the MDT satisfied? When is the MDT not satisfied? Examples?
  - When is the client satisfied? How do you know that your client is satisfied? Examples?
- During discharge:
  - What happens?
  - Who are involved? In which manner?
  - Which information from the client do you need?
  - Can you explain with some examples what goes well? And what does not go well?
  - When is the MDT satisfied? When is the MDT not satisfied? Examples?
  - When is the client satisfied? How do you know that your client is satisfied? Examples?
  - Can you explain the difference of discharge to home or to a nursing home?
- How do you define good quality of GRC? How do older adults define good quality of care? What is crucial in the GRC caring process?
- When is a rehabilitation successful? And, for whom is the rehabilitation successful?

What is the starting point for a successful rehabilitation?

- What is the strength of your GRC approach?
  - What works well?
  - What can be improved? How?
- What is the focus of the rehabilitation?
  - Which activities are necessary?
  - Which activities are meaningful?
  - To what extent are meaningful activities provided during intramural rehabilitation?
  - If you were the client, what would you like to achieve during the rehabilitaton (what would be your wish)? And what can you expect from the rehabilitation? Are there differences between the expectations and wishes?
- According to you, what should be important elements of the evaluation instrument?
  - What are facilitating factors for using the instrument?
  - What are barriers for using the instrument?

**Closing:**

- Thank the participant
- After this interview, how do you reflect on your job? Did you get other/new insights?
- Ask if the participant has any additions, something we didn’t discuss but what might be relevant
- Explain what’s next
- Discuss expert meeting
- Ask if the participant has any questions
