# Supplementary material for: Development of an evaluation tool for geriatric rehabilitation care
Source: BMC Geriatr. 2019 Aug 2;19:206. doi: 10.1186/s12877-019-1213-0 (PMC6679545; doi:10.1186/s12877-019-1213-0)
Supplement: Supplementary file 2 — Agenda expert meeting including topics to be discussed. (DOCX 15 kb) [file 12877_2019_1213_MOESM2_ESM.docx]

**Additional file 2: Agenda expert meeting including topics to be discussed:**

1. Become acquainted with the other respondents – 20 min

- All respondents mention a positive example of her GRC

1. Explain the aim of the study and of the expert meeting – 5 min
2. Share the results of the individual interviews – 25 min

- Check whether respondents recognize the results

1. Inspire each other: sharing positive examples – 5 min

- During the individual interviews respondents shared some examples that support the GRC quality and might inspire GRC professionals of other organizations

1. Energizer – 5 min
2. Results in relation to realist evaluation – 10 min

- Explain the theory of realist evaluation
- Discuss the mechanisms, contexts and outcomes found in the individual interviews

1. Break – 15 min
2. Start concretizing and defining each mechanism – 30 min

- The eight mechanisms were written down on A0-flaps
- In pairs, respondents work on specifying each mechanism, as a starting point for the definition of each mechanism
- Wrap up plenary

1. Closing with energizer – 5 min
